# Massive gene swamping among cheese-making *Penicillium* fungi

**DOI:** 10.15698/mic2014.01.135

**Published:** 2014-03-03

**Authors:** Jeanne Ropars, Gabriela Aguileta, Damien M. de Vienne, Tatiana Giraud

**Affiliations:** 1 Univ Paris-Sud, Ecologie, Systématique et Evolution, UMR8079, 91405 Orsay, France.; 2 CNRS, Ecologie, Systématique et Evolution, UMR8079, 91405 Orsay, France.; 3 Center for Genomic Regulation (CRG), Dr, Aiguader 88, 08003 Barcelona, Spain.; 4 CNRS, UMR 5558, Laboratoire de Biométrie et Biologie Evolutive.; 5 Université de Lyon; Université Lyon 1; CNRS; UMR 5558, Laboratoire de Biométrie et Biologie Evolutive.

**Keywords:** domestication, food, adaptive divergence, Saccharomyces

## Abstract

Horizontal gene transfers (HGT), *i.e.*, the transmission of genetic material between species not directly attributable to meiotic gene exchange, have long been acknowledged as a major driver of prokaryotic evolution and is increasingly recognized as an important source of adaptation in eukaryotes. In fungi in particular, many convincing examples of HGT have been reported to confer selective advantages on the recipient fungal host, either promoting fungal pathogenicity on plants or increasing their toxicity by the acquisition of secondary metabolic clusters, resulting in adaptation to new niches and in some cases eventually even in speciation. These horizontal gene transfers involve single genes, complete metabolic pathways or even entire chromosomes. A recent study has uncovered multiple recent horizontal transfers of a 575 kb genomic island in cheese *Penicillium *fungi, representing ca. 2% of the *Penicillium roqueforti*’s genome, that may confer selective advantage in the competing cheese environment where bacteria and fungi occur. Novel phylogenomic methods are being developed, revealing massive HGT among fungi. Altogether, these recent studies indicate that HGT is a crucial mechanism of rapid adaptation, even among eukaryotes.

A well-documented example of pathogenicity acquisition in fungi involves the recent transfer of the toxin-coding gene ToxA, from *Stagonospora nodorum*, a fungus pathogen of wheat, to *Pyrenophora tritici-repentis*, a distant fungal species having thereby acquired the ability to infect wheat. *Pyrenophora* has also acquired numerous virulence factors from bacteria, for instance involved in plant cell wall degradation or interfering with the plant defense response. HGTs have been frequent between plant pathogenic fungi and oomycetes, two phylogenetically very distant lineages in the tree of life while being similar in morphology and lifestyle. HGTs can also involve entire gene clusters. For instance, secondary metabolites involved in nitrate assimilation have been transferred horizontally in *Trichoderma reesi*, a fungus used in industry for its ability of cellulose hydrolysis. The wine yeast *Saccharomyces cerevisiae* acquired the ability to cope with the harsh conditions imposed by wine fermentation through HGT. Even whole chromosomes conferring pathogenicity can be horizontally transferred, such as in *Fusarium oxysporum* that gained several virulence-related proteins through HGT and *Alternaria alternata*, some strains of which acquired a small extra chromosome allowing them to infect tomato plants. HGT thus appears as an important mechanism promoting rapid adaptation to extreme environments, including man-made products, where very specific and homogenous conditions prevail. We thus unknowingly benefit from such advantageous effects of HGT every time we use fungi in the elaboration of wine, cheese, drinks and a large set of other industrial processes. Similar to HGTs in plants, which have been implicated in land colonization, HGTs in fungi might also have been involved in the transition to terrestrial environment.

A recent genomic comparison of two newly sequenced cheese-making *Penicillium*, *P. roqueforti *and *P. camemberti, *with the penicillin-producer *P. rubens* revealed several stretches of 100% nucleotidic identity that contrasted with the 85-90% of identity normally found between *Penicillium *species*. *In *P. roqueforti*, all these sequences clustered together within a single 575kb genomic region - called* Wallaby -* whereas they were fragmented and scattered at non-homologous loci in *P. camemberti* and* P. rubens *(Figure 1). Interestingly, the screening of a large collection of *Penicillium* strains and species coming from various environments, e.g. cheese, other type of food and natural environments, revealed that *Wallaby* was exclusively detected in *Penicillium *strains from food environments. All amplicons, obtained from hundreds of strains belonging to dozens of species, lacked variability, with even no synonymous substitutions, indicating that *Wallaby *was not an ancestral character and that the donor species was not in the collection. Nevertheless, the tetranucleotide composition of *Wallaby *indicated that the donor species of *Wallaby *most likely belonged to the *Penicillium* clade. Altogether, the 100% identity of *Wallaby *between several *Penicillium *species at different genomic loci*, *indicates that the transfer of *Wallaby* occurred several times and very recently, within a few years.

**Figure 1:**
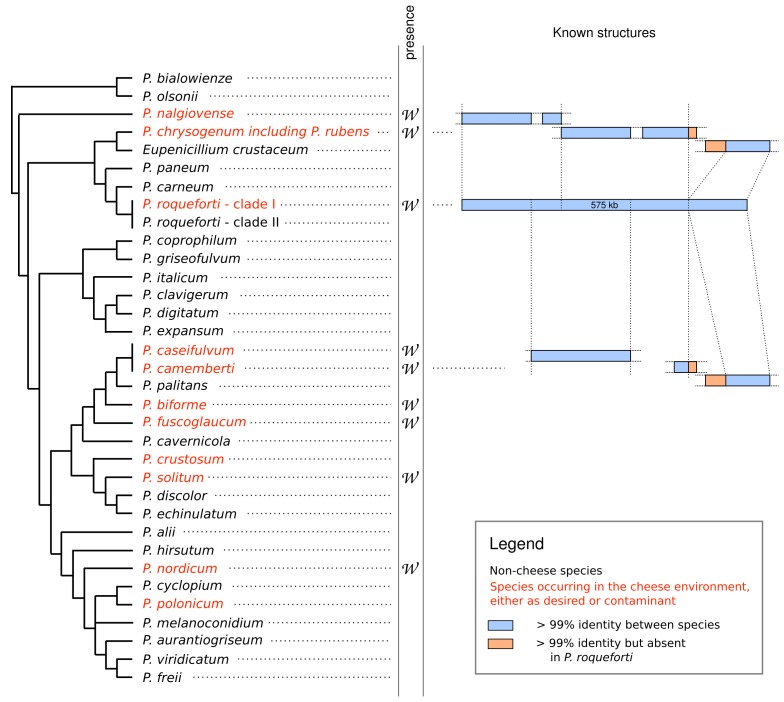
FIGURE 1: Phylogeny of *Penicillium *species screened by PCR for the presence of *Wallaby.* Several species occurring in the cheese environment (in red), either as desired or contaminant species, carry *Wallaby. *The structure of *Wallaby *is represented on the right hand side: the 575 Kb genomic island is in a single block in *P. roqueforti *(blue bar in the middle) while it is fragmented and located in non-homologous regions in *P. camemberti *(below) and *P. rubens *(above).

The mechanism by which *Wallaby *was transferred from a species to another remains to be discovered. In fungi, and in particular in *Penicillium*, anastomoses (i.e. somatic fusions of mycelia) can occur and lead to horizontal gene transfer. At the genetic scale, no transposable elements or duplications were found around *Wallaby*. In *P. roqueforti* however*, *flanking regions of *Wallaby *may be a hot spot of DNA insertions, as other unrelated fragments were inserted in other species at this specific locus.

The reason for such massive gene transfers among *Penicillium* cheese making fungi may be that *Wallaby* provides a competitive advantage in the nutrient-rich cheese environment. It indeed encompasses, among its 250 predicted genes, some that are likely to be involved in competition with microorganisms, such as the *Penicillium *antifungal protein PAF with a demonstrated role in antagonistic interactions with other microorganisms.

This specific horizontal transfer of a large DNA fragment in cheese-making fungi, along with numerous convincing examples published in the last ten years (see above), further highlight the importance of HGT in the evolution of fungi as a crucial mechanism of rapid adaptation, and in particular in human-made environments. It also suggests that HGTs among fungi may even be more frequent than currently recognized.

The genomes of more than 220 fungal species have been sequenced so far, rendering large-scale phylogenomic methods of HGT detection possible. Such methods are becoming extremely powerful, by taking into account not only transfer events, but also duplications and losses, allowing reconstructing complete evolutionary scenarios of each gene family and accurate detection of convincing cases of transfers. To date however, few large scale studies have been performed for estimating the amount of HGT in fungi. In one such study involving 60 genomes, more than 700 HGT events have been detected, all of prokaryotic origin. The observed bias in the identity of donors (all prokaryotes) may only be methodological: It is easier to detect transfers from prokaryotes to eukaryotes than within eukaryotes, because the evolutionary distance between donor and receptor is high and because many more prokaryotic than eukaryotic genomes are available. HGTs are however thought to occur more easily between more closely related species, because of their similar genomic environments. The estimated number of 700 HGTs acquired is therefore certainly largely underestimated. The *Wallaby* transfer recently described should be one of many fungi-to-fungi transfers detectable in large scale analyses of fungal genomes.

Another important aspect of the currently emerging studies of HGTs is the idea that gene transfers can be a very powerful tool in evolutionary studies. Indeed, they can be used for (i) rooting species trees, by minimizing the number of transfers detected, (ii) performing relative dating of speciation events in trees (transfers can only occur between contemporaneous species) or (iii) detecting extinct and unknown species or clades, in cases where species carry some genes that have been acquired through HGT from species that became extinct. In the next years, all these aspects of the large-scale study of HGT in fungi will contribute to a better definition of the Fungal Tree of Life, but also to make us aware of the risks of undesired escapes of genes introduced in domesticated species.

